# Genomic Features of an MDR *Escherichia coli* ST5506 Harboring an IncHI2/In*229*/*bla*_CTX-M-2_ Array Isolated from a Migratory Black Skimmer

**DOI:** 10.3390/pathogens13010063

**Published:** 2024-01-09

**Authors:** Quézia Moura, Miriam R. Fernandes, Fábio P. Sellera, Brenda Cardoso, Cristiane L. Nascimento, Gustavo H. P. Dutra, Nilton Lincopan

**Affiliations:** 1 Federal Institute of Espírito Santo, Vila Velha 29106-010, Brazil; 2 Postgraduate Program in Infectious Diseases, Federal University of Espírito Santo, Vitória 29047-105, Brazil; 3 Laboratory of Integrative Cancer Immunology, Center for Cancer Research, National Cancer Institute, National Institutes of Health, Bethesda, MD 20892-9760, USA; fernandes.miriamr@gmail.com; 4 Department of Internal Medicine, School of Veterinary Medicine and Animal Science, University of São Paulo, São Paulo 05508-270, Brazil; fsellera@usp.br; 5 School of Veterinary Medicine, Metropolitan University of Santos, Santos 11045-002, Brazil; 6 Department of Microbiology, Institute of Biomedical Sciences, University of São Paulo, São Paulo 05508-000, Brazil; brenda.cardoso@usp.br; 7 One Health Brazilian Resistance Project (OneBR), São Paulo 05508-000, Brazil; 8 Veterinary Unit of Santos Aquarium, Santos 11030-600, Brazil; cristianelassalvia@hotmail.com (C.L.N.); gustavodutra@santos.sp.gov.br (G.H.P.D.); 9 Department of Clinical Analysis, Faculty of Pharmacy, University of São Paulo, São Paulo 05508-000, Brazil

**Keywords:** ESBL, heavy metals, biocides, seabirds, wildlife, whole-genome sequencing

## Abstract

Migratory birds have contributed to the dissemination of multidrug-resistant (MDR) bacteria across the continents. A CTX-M-2-producing *Escherichia coli* was isolated from a black skimmer (*Rynchops niger*) in Southeast Brazil. The whole genome was sequenced using the Illumina NextSeq platform and de novo assembled by CLC. Bioinformatic analyses were carried out using tools from the Center for Genomic Epidemiology. The genome size was estimated at 4.9 Mb, with 4790 coding sequences. A wide resistome was detected, with genes encoding resistance to several clinically significant antimicrobials, heavy metals, and biocides. The *bla*_CTX-M-2_ gene was inserted in an In*229* class 1 integron inside a ∆Tn*As3* transposon located in an IncHI2/ST2 plasmid. The strain was assigned to ST5506, CH type *fumC19/fimH32*, serotype O8:K87, and phylogroup B1. Virulence genes associated with survival in acid conditions, increased serum survival, and adherence were also identified. These data highlight the role of migratory seabirds as reservoirs and carriers of antimicrobial resistance determinants and can help to elucidate the antimicrobial resistance dynamics under a One Health perspective.

## 1. Introduction

Extended-spectrum β-lactamase (ESBL)-producing Enterobacterales have been considered one of the greatest threats to human health, being listed as critical priority pathogens by the World Health Organization (WHO) [1]. Despite greater emphasis being placed on the human clinical sphere, the One Health approach draws attention to the need for studies involving other ecological spheres, given the interdependence among humans, animals, and the environment [2]. Dissemination of antimicrobial resistance between these different sectors can occur through direct transmission of resistant bacteria or through genetic exchanges, mainly by plasmids and other mobile genetic elements, as integrons and transposons [3]. In this regard, whole-genome sequencing can be a valuable tool for surveillance of antimicrobial resistance, as it allows a deep understanding of the genetic basis of resistance mechanisms, evolution, and dissemination [4].

Since wild animals can also be exposed to human-associated multidrug-resistant (MDR) pathogens, there is a global trend for monitoring the dissemination of medically relevant bacteria in wildlife populations [5]. Recently, migratory birds have been recognized as important reservoirs and vectors for spreading ESBL-producers across the globe [5,6]. When contacting contaminated environments, these animals can incorporate antimicrobial resistant bacteria, as ESBL-producing *Escherichia coli*, in the gut microbiota; then, it can be spread over distant geographic locations, according to the migratory route [7].

Black skimmers (*Rynchops niger*) are widespread piscivorous seabirds that inhabit sandy shorelines, being commonly found in the Nearctic and Neotropical regions [8]. Due to their migration behavior, they appear seasonally in large numbers in both the Pacific and Atlantic coasts of South America and are frequently observed in bays, estuaries, beaches, and shallow lagoons, where they prey on populations of small fish [8]. In this study, we aimed to analyze the genomic features of an MDR ESBL-producing *E. coli* strain recovered from the gut microbiota of a migratory black skimmer.

## 2. Materials and Methods

In March 2016, as part of a surveillance study on antimicrobial-resistant Gram-negative bacteria among wild animals, a cloacal swab sample was collected from a black skimmer (*R. niger*), at the admission time to a wildlife rehabilitation center. The bird was rescued by firefighters at a beach on the southeast coast of Brazil (−23.985143, −46.309956) and presented a fracture of the right humerus. Until the sample collection time, no medication had been administered to the bird. The sample was inoculated on MacConkey agar plates supplemented with ceftriaxone (2 μg/mL), colistin (2 μg/mL) and ciprofloxacin (1 μg/mL) (Sigma-Aldrich, St. Louis, MO, USA), for screening of bacteria resistant to cephalosporins, polymyxins, and fluoroquinolones, respectively. Agar plates were incubated for 18–24 h, at 35 °C. The bacterial isolate was identified by matrix-assisted laser desorption/ionization time-of-flight mass spectrometry (MALDI-TOF). Antimicrobial susceptibility was determined by disk diffusion and E-test methods [9], and the colistin susceptibility test was performed by the broth microdilution method, according to the recommendations of the Clinical and Laboratory Standards Institute (CLSI) [9]. *E. coli* 25922 was used as the quality control strain.

Genomic DNA extraction was carried out using PureLink™ Quick Gel Extraction Kit (Life Technologies, Carlsbad, CA, USA), and the DNA concentration was measured using a Qubit^®^ 2.0 fluorometer (Life Technologies, Carlsbad, CA, USA). The genomic library was built using a Nextera XT DNA Library Preparation Kit (Illumina Inc., Cambridge, UK), according to the manufacturer’s instructions. The whole genome sequencing was performed on an Illumina NextSeq platform, using paired-end reads (150 bp). Reads with a PHRED quality score below 20 were discarded, and adapters were trimmed using TrimGalore v0.6.5 (https://github.com/FelixKrueger/TrimGalore, accessed on 30 June 2023). De novo genome assembly was performed using CLC Genomics Workbench v. 11 (CLC Bio, Aarhus, Denmark), and the draft genome was annotated using the NCBI Prokaryotic Annotation Pipeline [PGAP (https://www.ncbi.nlm.nih.gov/genome/annotation_prok/, accessed on 17 October 2023)].

Acquired antimicrobial resistance genes (ARGs) were evaluated by ResFinder 4.1 [10], and the genetic context of some ARGs was analyzed by using Geneious Prime^®^ 2022.0.1 (Biomatters, Auckland, New Zealand). Insertion sequences and transposons were identified by ISFinder (https://www-is.biotoul.fr/index.php, accessed on 30 June 2023). The BLASTn tool (https://blast.ncbi.nlm.nih.gov/Blast.cgi, accessed on 30 June 2023) was used for identification of similar genetic environments, and the comparative analysis of these genetic structures was displayed by using Easyfig version 2.1 (http://mjsull.github.io/Easyfig/, accessed on 17 October 2023). Plasmid incompatibility groups and pMLST were predicted with PlasmidFinder 2.1 [11] and pMLST 2.0 [11], respectively. Heavy metal and biocide resistance genes were detected using the BacMet database [12]. MLST, CH type, serotype, and virulence genes were predicted with MLST 2.0 [13], CHTyper 1.0 [14], SerotypeFinder 2.0 [15], and VirulenceFinder 2.0 [16], respectively. *E. coli* phylogroup was determined through the ClermonTyping online tool [17]. A ≥90% identity threshold was used for all predicted genes. Genomic comparison of ICBTMS1 with other genomes from the same ST—available from EnteroBase (https://enterobase.warwick.ac.uk/species/index/ecoli, accessed on 17 October 2023)—was performed using BRIG 0.95 software [18].

No ethical approval was required for this study. Biological sample collection was authorized by the Authorization System and Information on Biodiversity (SISBIO license number 55804–2).

## 3. Results and Discussion

A single *E. coli* strain (ICBTMS1) was recovered from the MacConkey agar plate supplemented with ceftriaxone. No growth was observed on the other agar plates. ICBTMS1 displayed an MDR profile to amoxicillin/clavulanic acid, cefotaxime, ceftazidime, cefepime, ceftriaxone (MIC ≥ 32 μg/mL), aztreonam, gentamicin, trimethoprim/sulfamethoxazole, and tetracycline. Otherwise, it was susceptible to cefoxitin, ciprofloxacin, amikacin, imipenem, ertapenem, meropenem, and colistin. *E. coli* 25922 presented susceptibility results within acceptable quality control ranges.

A total of 6,161,326 reads, assembled into 154 contigs, was produced with 123x coverage and a G+C content of 48.4%. The genome size was calculated at 4,968,129 bp, comprising 4790 coding sequences (CDS), 2 rRNAs, 44 tRNAs, 9 ncRNAs, 134 pseudogenes, and 2 CRISPR arrays.

Resistome analysis detected the presence of antimicrobial genes conferring resistance to β-lactams (*bla*_CTX-M-2_, *bla*_TEM-1C_), aminoglycosides [*aac(3)-VIa*, *aadA1*, *aph(3′)-Ia*], sulfamethoxazole (*sul1*), trimethoprim (*dfrA27*), and tetracycline (*tetA*) (Table 1).

**Figure 1:**
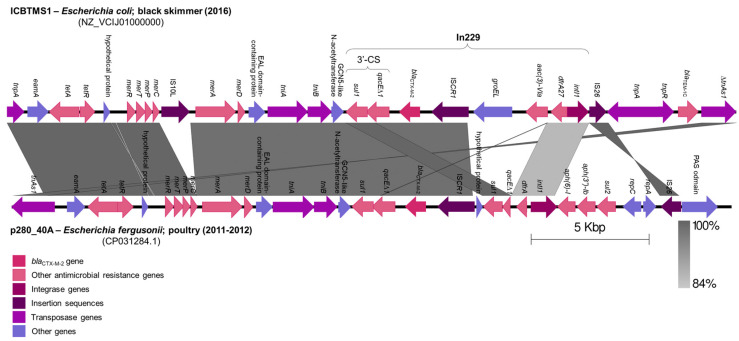
Comparison of genomic structures from IncHI2/ST2 plasmids harboring the *bla*_CTX-M-2_ gene. A fragment of the IncHI2/ST2 plasmid from the ICBTMS1 *E. coli* strain, isolated from the black skimmer (2016), was compared with a fragment of an IncHI2/ST2 plasmid from an *E. fergusonii* isolated from poultry (2011–2012), using Easyfig version 2.1 (http://mjsull.github.io/Easyfig/, accessed on 17 October 2023). Regions of homology are marked by gray shading.

The *bla*_CTX-M-2_ gene was inserted in an In*229* class 1 integron by an IS*CR1*, downstream of the 3′-conserved segment (3′-CS), inside a ∆Tn*As3* transposon (Figure 1). This genomic structure was located in an IncHI2/ST2 plasmid and showed 73% coverage and 99.71% identity with a 29,941 bp fragment of an IncHI2/ST2 plasmid (CP031284.1) from an *Escherichia fergusonii* isolated from poultry, in São Paulo State, Brazil, between 2011 and 2012 [19] (Figure 1). Indeed, IncHI2/ST2 plasmids have been previously described in Brazil, in *Salmonella* spp. isolates from poultry, also carrying the *bla*_CTX-M-2_ gene [20,21]. These data infer the occurrence of genetic exchanges among bacterial strains from wild birds and poultry, as already suggested by previous studies [22,23].

ICBTMS1 also carried a variety of genes conferring resistance to heavy metals, as arsenical (*arsB*), cadmium (*ychH* and *yhcN*), chromate (*yieF*), cobalt (*corABD*), copper (*cueO*, *cusARS*, and *cutCEF*), iron/manganese (*sitABCD*), magnesium (*mgtA*), manganese (*mntHPR*), mercury (*merRTPCAD*), molybdate (*modABCE*), nickel (*nikABCDER*), silver (*silAR*), tellurite (*tehAB*), tellurium (*terACDEWZ*), zinc/tellurium (*pitA*), and zinc (*zinT*, *zitB*, *zntAR*, *znuAB*, *zraR*, *zupT*, and *zur*), in addition to biocide resistance genes (*ostA*, *oxyRkp*, *qacE∆1*, and *sugE*) (Table 1). Occurrence of these genes in wild animals and the environment is suggestive of heavy metal contamination [24]. Some studies, carried out in the same region where the black skimmer was found, have detected high levels of heavy metals in estuary sediment samples [25,26,27]. In addition, heavy-metal-resistant bacteria have been isolated from wild fish and shrimp in that area [28,29]. The presence of heavy metals in animals and the environment can boost bacterial resistance to both heavy metals and antimicrobials, through co- or cross-selection mechanisms [30].

**Figure 2:**
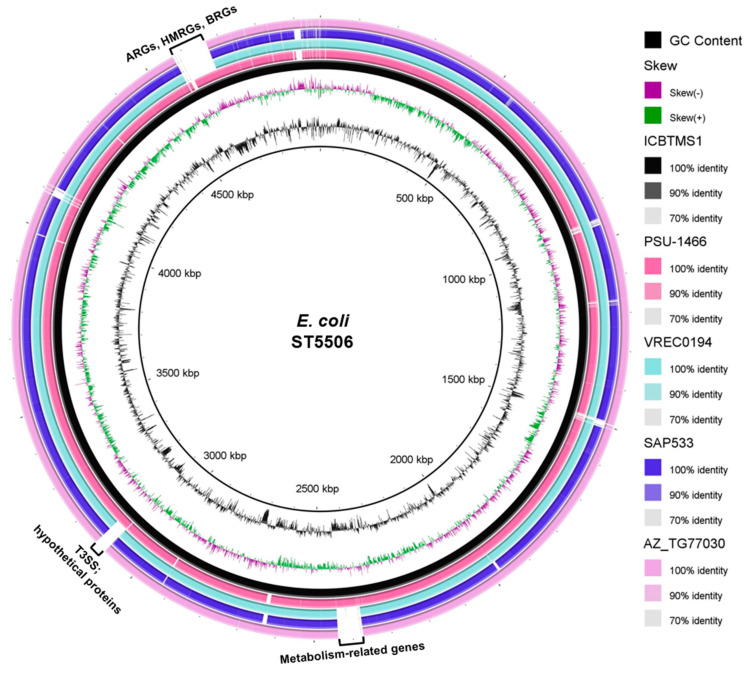
Genomic comparison of ICBTMS1 against four ST5506 *E. coli* genomes, made by using the BRIG software (http://brig.sourceforge.net/, accessed on 12 July 2023). The innermost rings depict GC content (black) and GC skew (purple/green), followed by concentric rings of ICBTMS1 (black; accession number: NZ_VCIJ01000000), PSU–1466 (pink; accession number: AATPIY000000000.1), VREC0194 (light blue; accession number: DADKSK000000000.1), SAP533 (purple; accession number: SAMEA3697202), and AZ_TG77030 (light pink; accession number: AATISO000000000.1), colored according to BLASTn identity with 90% and 70% as the upper and lower identity thresholds, respectively. Gapped regions indicate the absence or low similarity among the genomes. ARGs: antimicrobial resistance genes; HMRGs: heavy metal resistance genes; BRGs: biocide resistance genes; T3SS: type III secretion system.

The strain was assigned to the ST5506, which has already been reported in animal and environmental strains (https://enterobase.warwick.ac.uk/species/index/ecoli, accessed on 12 July 2023): the strain PSU-1466 (accession number: AATPIY000000000.1), isolated from turkey, in the USA, in 1990; the strain AZ_TG77030 (accession number: AATISO000000000.1), isolated from chicken meat, in the USA, in 2014; the strain VREC0194 (accession number: DADKSK000000000.1), isolated from water, in England, in 2015; and the strain SAP533 (accession number: SAMEA3697202), isolated in England, in 2015 (unknown source). When compared with the other genomes from ST5506, ICBTMS1 showed differences mainly in the regions of antimicrobial, heavy metal, and biocide resistance genes; the type three secretion system (T3SS); hypothetical proteins; and metabolism-related genes (Figure 2). A higher genetic similarity was found with the strain PSU-1466 (AATPIY000000000.1) (Figure 2). Additionally, the strain belonged to the O8:K87 serotype, known to be an important animal pathogen [31,32]; CH type *fumC19*/*fimH32*; and the commensal phylogroup B1 [33]. Virulence genes related to survival in acid conditions (*gad*), increased serum survival (*iss*), and adherence (*lpfA* and *papC*) were detected, as well.

In summary, we report an international *E. coli* clone, isolated from a migratory seabird, carrying a wide resistome against clinically significant antimicrobials, heavy metals, and biocides, which should be considered a critical epidemiological issue. The broad resistome identified in this WHO critical priority pathogen is likely related to an environment impacted by anthropogenic activities [34]. In addition, these data highlight the importance of monitoring migratory seabirds as reservoirs and carriers of such bacteria and genetic determinants and their role as sentinels of ecosystem health [35]. Last but not least, this study provides valuable genomic information that might be useful in understanding the antimicrobial resistance dynamics under a One Health perspective.

## Figures and Tables

**Table 1:** Phenotypic and genomic data from *Escherichia coli* strain ICBTMS1.

Strain	ICBTMS1
AMR profile ^1^	AMC, CAZ, CRO, CTX, FEP, ATM, GEN, SXT, TET
ST/CC	5506
CH type	*fumC19*/*fimH32*
Serotype	O8:K87
*E. coli* phylogroup	B1
Resistome	
β-lactams	*bla*_CTX-M-2_, *bla*_TEM-1C_
Aminoglycosides	*aac(3)-VIa*, *aadA1*, *aph(3′)-Ia*
Sulfamethoxazole	*sul1*
Trimethoprim	*dfrA27*
Tetracycline	*tetA*
Heavy metals	*arsB*, *corABD*, *cueO*, *cusARS*, *cutCEF*, *merET*, *mgtA*, *mntHPR*, *modABCE*, *nikABCDER*, *pitA*, *silAR*, *sitABCD*, *tehAB*, *terACDEWZ*, *ychH*, *yieF*, *zinT*, *zitB*, *zntAR*, *znuAB*, *zraR*, *zupT*, *zur*
Biocides	*ostA*, *oxyRkp*, *qacE∆1*, *sugE*
Plasmid	IncHI2 (ST2)
Virulence genes	*gad*, *iss*, *lpfA*, *papC*
GenBank accession number	NZ_VCIJ01000000

^1^ AMC: amoxicillin–clavulanic acid; CAZ: ceftazidime; CRO: ceftriaxone; CTX: cefotaxime; FEP: cefepime; ATM: aztreonam; GEN: gentamicin; SXT: trimethoprim–sulfamethoxazole; TET: tetracycline.

## Data Availability

This Whole Genome Shotgun project has been deposited at DDBJ/ENA/GenBank under accession number NZ_VCIJ01000000. The version described in this paper is the first version. Additionally, data are also available at the OneBR platform (http://onehealthbr.com, accessed on 1 January 2024), under the ID number ONE134.
